# Early maternal perceived stress and children’s BMI: longitudinal impact and influencing factors

**DOI:** 10.1186/s12889-018-6110-5

**Published:** 2018-10-30

**Authors:** Beate Leppert, Kristin M. Junge, Stefan Röder, Michael Borte, Gabriele I. Stangl, Rosalind J. Wright, Anja Hilbert, Irina Lehmann, Saskia Trump

**Affiliations:** 1 Department of Environmental Immunology, Helmholtz Centre for Environmental Research (UFZ), Leipzig, Germany; 2 Children’s Hospital, Municipal Hospital “St. Georg”, Leipzig, Germany; 3 Institute of Agricultural and Nutritional Sciences, Martin Luther University Halle-Wittenberg, Halle (Saale), Germany; 4 Competence Cluster for Nutrition and Cardiovascular Health (nutriCARD) Halle-Jena, Leipzig, Germany; 5 Department of Pediatrics, Kravis Children’s Hospital, Institute for Exposomic Research, Icahn School of Medicine at Mount Sinai, New York, USA; 6 Integrated Research and Treatment Center AdiposityDiseases, Department of Medical Psychology and Medical Sociology, Department of Psychosomatic Medicine and Psychotherapy, University of Leipzig Medical Center, Leipzig, Germany; 7 Charité - Universitätsmedizin Berlin, corporate member of Freie Universität Berlin, Humboldt-Universität zu Berlin, and Berlin Institute of Health, Kapelle Ufer 2, 10177 Berlin, Germany

**Keywords:** Stress dimensions, Perceived stress, Weight development, Stressor, Infant, Preschool children

## Abstract

**Background:**

Maternal perceived stress has been discussed to contribute to the development of childhood overweight. Our aim was to investigate the longitudinal relationship of early maternal perceived stress and BMI z-scores in preschool children (≤ five years).

**Methods:**

A longitudinal analysis was conducted in 498 mother-child pairs of the German prospective birth cohort LINA with information on maternal perceived stress during pregnancy, one and two years after birth. BMI z-scores were based on annual measurements of children’s weight/height and calculated based on WHO reference data. General estimation equations were applied to evaluate the impact of maternal stress on children’s longitudinal BMI z-scores. Potential stressors contributing to the perceived stress of the mother were assessed by linear regression models. Using mediation analyses we evaluated the relationship between stressors, maternal perceived stress, and children’s BMI z-score development.

**Results:**

Postnatal maternal stress during the first year after birth had a positive longitudinal relationship with children’s BMI z-scores up to the age of five years. Gender-stratified analyses revealed that only girls showed this positive association while boy’s BMI z-scores were unaffected by maternal stress. We identified three neighborhood strains and two socio-demographic factors, which contributed to the maternal perceived stress level. Stressors themselves did not directly affect girl’s BMI z-scores but rather mediated their effect through the perceived stress level.

**Conclusions:**

While different stressors contribute to maternal stress, the perceived stress level - rather than the stressors themselves - is strongly positively associated with BMI z-score development in girls.

**Electronic supplementary material:**

The online version of this article (10.1186/s12889-018-6110-5) contains supplementary material, which is available to authorized users.

## Background

Overweight and obesity prevalence, especially among preschool children, has risen dramatically world-wide over the last decades affecting 6.1% of children under five years of age in 2016 [1]. Recent data from the KiGGS study shows that in Germany this fraction is even higher with 9.5% of children age two-six being overweight, of which 2.8% are classified as obese [2]. This is particularly concerning as most of these children will remain overweight in adolescence and adulthood, increasing their risk for co-morbidities like cardiovascular diseases or type 2 diabetes mellitus [3, 4].

Unhealthy diet and physical inactivity have been described as the main risk factors contributing to obesity development [5]. Consequently, child obesity intervention and prevention studies are mainly focusing on implementing changes in eating behavior and physical activity. However, most of these studies failed to reach long-term effects [6, 7], suggesting that other factors such as the living environment and parental behavior play an important role in the context of children’s overweight development. Among others, psychological aspects like early infant parental distress [8] as well as maternal depression [9] emerged as potential factors promoting children’s overweight.

Accumulating research shows the wide-ranging consequences of maternal stress on children’s health, which among others include an increased risk for behavioral problems, asthma, reduced birth weight, and an increased risk for becoming overweight [10–12]. Perceived stress is the individual perception about the stressfulness of life and the ability to handle such stress, which can be influenced by a variety of sources. These so-called stressors include socioeconomic disadvantages as well as recent life events like divorce/separation that can ultimately lead to stress-related physiological dysregulations [13, 14]. In this context maternal stress during pregnancy is known to alter signaling in the hypothalamic-pituitary-axis (HPA) exposing the developing fetus to an excess of glucocorticoids [15], one of the mechanisms discussed to contribute to prenatal growth restriction [16, 17] and an accelerated catch-up-growth increasing the risk for obesity in children’s later life [18–20]. While in the prenatal phase the physiological stress response of the mother can directly affect the child, in the postnatal period - as assessed here in the first two years after birth - the impact of maternal stress on parenting behavior and mother-child interactions become important. There is evidence that changes in feeding styles and practices [21] due to parental or maternal stress can have a significant impact on children’s food composition and energy intake [22]. Especially maternal stress becomes important as mothers often spend significantly more time in direct interaction with the child compared to the fathers. For example higher infant energy intake and increased consumption of breads and cereals during the first six month after birth have been described in association with maternal stress or depression [23], as has children’s reduced consumption of fruits and vegetables [24, 25]. Moreover, stress perceived by children themselves seems to alter their energy intake and food selection with a preference for sweet and high fat foods [25].

The majority of studies conducted so far focused on prenatal or postnatal stress exclusively, while longitudinal maternal stress assessments in relation to children’s weight development are rare. As recently reviewed by *Tate et al*. [12] and *O’Connor et al*. [26] most of these studies only used either longitudinal information on maternal stress or children’s overweight development. Therefore, our aim was to investigate the association between maternal stress and body mass index (BMI) trajectories in children in a longitudinal manner, including maternal stress evaluations from pregnancy until children’s age of two years and annual weight assessments of the children up to the age of five years. We complement our study by analyzing which stressors might contribute to the perceived maternal stress level and therefore might have a potential impact also on children’s weight development throughout the years. It has been discussed that the living environment including noise exposure and an unsecure living environment influence weight development in adults [27–29]. Since data on the effects of such stressors on children is sparse [30] we included not only the socioeconomic status of the study participants, but also factors characterizing their living environment such as traffic or residential noise in our analysis.

We hypothesize that with our longitudinal qualitative stress assessment we will be able to identify a time window, in which the child is particularly vulnerable to maternal stress and that such stress exposure experienced in this time window might have a long-lasting effect on the development of overweight in the child.

## Methods

### Study characteristics

The German prospective birth cohort LINA (Lifestyle and Environmental Factors and their Influence on Newborns Allergy risk) recruited 629 mother-child pairs at pregnancy (36^th^ week of gestation) during May 2006 and December 2008, as has been described in more detail elsewhere[31–33]. Lifestyle, housing, and environmental factors were assessed by questionnaires during pregnancy and annually thereafter. Stress questionnaires from three time points (pregnancy, age 1, age 2) together with information on gender, gestational week at delivery, mode of delivery, breastfeeding and prenatal environmental tobacco smoke exposure (ETS) were available for 498 mother-child pairs, which we defined as our analyzed sub-cohort (Table 1). All questionnaires were self-administered by the parents and participation in the study was voluntary. Written informed consent was obtained from all individual participants included in the study. The study was approved by the Ethics Committee of the University of Leipzig (file ref # 046-2006, 160-2008, 160b/2008, 144-10-31052010, 113-11-18042011).

**Table 1:** General study characteristics of the LINA cohort

	entire LINA cohort n (%), *n* = 629 ^a^	analyzed sub-cohort n (%), *n* = 498 ^a^	χ^2^-test
Gender			0.810
Male	330 (52.5)	253 (50.8)	
Female	299 (47.5)	245 (49.2)	
Week of gestation at birth			0.951
<37 weeks	25 (4.0)	16 (3.2)	
37-40 weeks	389 (61.8)	308 (61.8)	
> 40 weeks	214 (34.0)	174 (34.9)	
Mode of delivery			0.979
Spontaneous	471 (74.9)	387 (77.7)	
C-section	132 (21.0)	104 (20.9)	
Others	7 (1.1)	7 (1.4)	
Birth weight			0.996
< 3000g	123 (19.6)	92 (18.5)	
≥ 3000g – 3500g	242 (38.5)	195 (39.2)	
≥ 3500g – 4000g	192 (30.5)	151 (30.3)	
≥ 4000g	71 (11.3)	60 (12.0)	
Household members			0.887
2	33 (5.2)	26 (5.2)	
3	365 (56.6)	300 (60.2)	
≥4	203 (32.3)	196 (39.4)	
Breastfeeding			0.515
1.-3. month	112 (17.8)	87 (17.5)	
1.-6. month	268 (42.6)	166 (33.3)	
1.-12. month	254 (40.4)	226 (45.4)	
Parental education ^b^			0.697
Low	16 (2.5)	6 (1.2)	
Medium	144 (22.9)	101 (20.3)	
High	468 (74.4)	391 (78.5)	
Household income / month			0.648
< 2000€	240 (38.2)	172 (34.5)	
2000€ - 4000€	308 (49.0)	171 (34.3)	
> 4000€	42 (6.7)	35 (7.0)	
Separation/divorce ^c^			0.973
Yes	25 (4.0)	23 (4.6)	
No	169 (26.9)	158 (31.7)	
Prenatal ETS exposure ^d^			0.243^e^
Median [μg/g creatinine]	2.0	1.85	
< 25% , > 75%	0.8,5.6	0.75,4.95	

^a^n may be different from total n due to missing data

^b^Low = 8 yrs of schooling (‘Hauptschulabschluss`); medium = 10 yrs of schooling (`Mittlere Reife`); high = 12 yrs of schooling or more (`(Fach-)hochschulreife’)

^c^Parental separation/divorce in the last 3 years from children’s age 2 years

^d^*ETS* environmental tobacco smoke (urinary cotinine level at pregnancy)

^e^*p*-value derived from Student’s *T* test between group means

### Perceived maternal stress assessment

Maternal stress levels were assessed at 36^th^ weeks of gestation and at the one- and two-year follow-up using the 20-item reduced Perceived Stress Questionnaire (PSQ), a validated instrument by *Fliege et al*. [34, 35]. The PSQ is comprised of 5 items for each of the different stress dimensions “demands”, “tension”, “worries”, and “lack of joy”. All items were scored on a four-point scale according to the frequency of perception with one (hardly ever) to four (usually). The total stress score was derived as the mean of all 20 scored questions. The scores for each dimension were derived accordingly from the 5 dimension-specific questions [36]. Higher scores indicate higher stress levels.

### Anthropometric data

Children’s body weight and height up to the age of five years were obtained from annual clinical visits or from questionnaires of well-child exams (“U examination”). Child length was measured horizontally at birth and at the year 1 follow-up using an infantometer (“Dr. Keller II”). From year two onwards standing height was measured without shoes to the nearest 0.1 cm (“Dr. Keller I”). Body weight was measured to the nearest 0.1 kg, and BMI z-scores were calculated according to the WHO reference data [37] to adjust for child’s age and gender. The use of z-scores is recommended for several reasons. First, z-scores are calculated based on the distribution of the reference population (both the mean and the SD); thus, they reflect the reference distribution. Second, as standardized measures, BMI z-scores are comparable across age and sex. Third, a group of z-scores can be subject to summary statistics such as mean and SD and - even more importantly in our case - can be studied as a continuous variable. Children with BMI z-score < -1 were classified as underweight, children with a BMI z-score of -1 to <1 were classified as normal weight, and children with BMI z-scores ≥1 were classified as overweight.

### Assessment of stressors

We assessed the impact of several stressors evaluated by questionnaires on maternal stress perceived during the first year after birth. The stressors evaluated considered the neighborhood quality (living conditions, exposure to traffic or residential noise) and the socio-demographic factors (household income, parental educational level, number of household members, the age of the mother at birth, divorce/separation).

Information about the household income, the parental educational level and the number of household members (all children and adults in the household), were recorded once during pregnancy. Neighborhood quality was assessed each year from pregnancy onwards using the questions summarized in Additional file 1: Table S1. The information on divorce/separation was based on a retrospective assessment at year two with respect to the preceding three years, the exact time point of divorce/separation was not inquired.

### Statistical analysis

In all analyses except where explicitly stated otherwise, a *p*-values ≤ 0.05 was considered to be significant. Whenever Bonferroni-correction was applied the adjusted significant levels are indicated.

To test for potential differences in study characteristics of the entire cohort compared to the analyzed sub-cohort and to test for equal distribution of parameters in the gender-specific analyses chi-square tests were conducted. The maternal perceived stress scores were assessed by Spearman correlations and a repeated measurement analysis of variance (RANOVA) was performed to assess time-dependent changes.

Generalized estimating equation (GEE) models with unstructured correlation matrices were applied to assess the effect of maternal stress on children’s longitudinal BMI z-scores (birth to age five or age one to age five respectively). These models were calculated with BMI z-scores and maternal perceived stress scores as continuous variables and were adjusted for weight-related confounding parameters based on a literature review[38–40], namely: gestational week at delivery, mode of delivery, breastfeeding duration, exposure to environmental tobacco smoke (urinary cotinine level during pregnancy, as described in Table 1), and for gender if applicable. To test for gender differences in response to maternal perceived stress the GEE models were stratified accordingly.

A principal factor analysis with oblique rotation was conducted on 10 questionnaire items assessing neighborhood strains to extract potential underlying scales (Additional file 1: Table S1). Factors with an eigenvalue greater than 1 were chosen following Kaiser’s criterion. Scales were composed of variables with factor loadings greater 0.4. These extracted scales together with different socio-demographic-factors (household income, parental educational level, number of household members, age of the mother at birth, divorce/separation) were assessed by linear regression for their impact on maternal perceived stress and the different dimensions thereof.

Mediation analysis based on the PROCESS SPSS macro (release 2.16.3 [41]) was applied to test the hypothesis whether the stressors identified to contribute to the perceived maternal stress level affect children’s BMI z-score development directly or indirectly. For this purpose, children were categorized in being overweight (BMI z-score ≥ 1) ever in the first five years of life and compared to children never being overweight (BMI-z-scores <1) in this time window. Perceived stress scores and stressors were considered as continuous variables in these analyses. Confidence intervals were computed based on 5000 bootstrap samples.

General statistical analyses, regression- and GEE models were conducted using STATISTICA 12.0 for Windows (Dell Inc., USA) or IBM SPSS Statistics version 22 (IBM Corps., USA) respectively.

## Results

### Study characteristics

Our analyses were based on the sub-cohort of 498 mother-child pairs for which a complete PSQ assessment for pregnancy, year 1 and year 2 including all weight-related confounders were available. General study characteristics of the analyzed sub-cohort (*n*=498) and the entire LINA cohort (*n*=629) were equally distributed as shown in Table 1. In the analyzed sub-cohort the majority of children (68 %) started daycare in their second year of life.

### Perceived maternal stress assessment

Pre-and postnatal maternal stress levels were highly correlated with each other (Spearman correlation, birth vs. age 1: R= 0.60, *p*<10^-13^; age 1 vs. age 2: R= 0.69, *p*<10^-13^; birth vs. age 2: R= 0. 58 *p*<10^-13^). There was a statistically significant increase in the maternal perceived stress scores over time as determined by RANOVA (F = 3772, *p*<0.0005, partial eta-squared = 0.94). An overview of median, minimum, maximum, lower and upper quartiles of maternal stress scores over time is given in Table 2A.

**Table 2:** Descriptive statistics of (A) maternal perceived stress scores. Given are median, min, max, and quartile boundaries (*n*=498). (B) BMI z-score categories within the analyzed sub-cohort^a^

A - Stress score		median	min	max	<25 %	>75%
pregnancy		1.9	1.0	3.6	1.6	2.3
year 1		2.0	1.1	3.9	1.7	2.4
year 2		2.2	1.0	3.9	1.9	2.5
B - BMI z-score^a^		Year 1 *n* = 487(%)	Year 2 *n* = 456(%)	Year 3 *n* = 428(%)	Year 4 *n* = 394(%)	Year 5 *n* = 352(%)
< 0	Underweight	112 (23.0)	25 (5.5)	43 (10.0)	40 (10.2)	45 (12.8)
0 < 1	Normal weight	336 (69)	307 (67.3)	309 (72.2)	303 (76.9)	266 (75.6)
≥ 1	Overweight	39 (8.0)	124 (27.2)	76 (17.8)	51 (12.9)	41 (11.6)

^a^Categorization based on WHO-reference data

#### Anthropometric data

On average, about 15.5 % of the analyzed LINA children became overweight during the first five years of life, reaching highest percentages at year two and three. A summary of categorized BMI z-scores of preschool children up to the age of five years is given in Table 2B.

### Longitudinal association of maternal perceived stress and children’s BMI z-scores

The adjusted GEE model showed a significant longitudinal effect on children’s BMI z-scores until the age of 5 years only for maternal perceived stress assessed at year one (adj. β: 0.23, 95% CI (0.08-0.37), *p* = 0.002) (Table 3A, Additional file 1: Table S2). There was no effect of maternal stress during pregnancy or year two on children’s BMI z-scores. Neither weight- nor height- z-scores were affected by maternal stress (data not shown).

**Table 3:** Impact of maternal perceived stress levels on longitudinal BMI z-score development in preschool children (birth-age 5)

		β estimate^a^	95% CI	*p*-value
A - Maternal perceived stress at				
Pregnancy	(*n* = 498)	0.06	-0.07 – 0.20	0.372
Year 1	(*n* = 491)	**0.23**	**0.08** – **0.37**	**0.002**
Year 2	(*n* = 473)	0.09	-1.47 – 1.64	0.283
Pregnancy to year 2	(*n* = 473)	0.06	-0.01 – 0.12	0.078
B - Sex-stratified effect of maternal perceived stress at year 1				
Girls only	(*n* = 241)	**0.30**	**0.11** – **0.49**	**0.002**
Boys only	(*n* = 250)	0.10	-0.11 – 0.31	0.333

(A) Effects of maternal perceived stress during pregnancy, year 1 and year 2 (B) Sex disparity in susceptibility to maternal perceived stress at year 1. Significant associations are presented in bold (*p* ≤ 0.05)

^a^Estimates derived from general estimation equations *GEE* for BMI z-scores (birth to age 5) as dependent variable, adjusted for gestational week at delivery, mode of delivery, pregnancy cotinine levels and breastfeeding duration (not for pregnancy stress levels)

Stratifying the GEE model for gender revealed a positive association of BMI z-scores with maternal stress during the first year after birth only for of girl’s (adj. β: 0.30, 95% CI (0.11-0.49), *p* = 0.002; Table 3B, Additional file 1: Table S3), whereas no association was seen for boys. This gender-specific effect was not based on different study characteristics between boys and girls, as can be seen from Additional file 1: Table S4. As only maternal perceived stress during the first year after birth had an effect on BMI z-scores, we focused our further analyses on this early postnatal period.

### Influence of different stress dimensions on BMI z-score development

Median, minimum, maximum, lower (<25%) and upper quartiles (>75%) of the four maternal stress dimensions assessed by the PSQ (“demands”, “tension”, “worries” and “lack of joy”) are given in Additional file 1: Table S5. GEE models were applied to evaluate their association with children’s longitudinal BMI z-scores. Results of the adjusted models are summarized in Table 4. After Bonferroni-correction, the stress dimensions “tension”, “lack of joy”, and “demands” showed a significantly positive association with children’s BMI z-scores. Similar to the total stress score the different dimensions of maternal stress only showed an association to girl’s BMI z-scores. “Worries”, “lack of joy”, and “demands” were significantly associated with higher BMI z-scores in girls, “lack of joy” showed the best model fit (QIC).

**Table 4:** Effect of the different stress dimensions on the longitudinal BMI z-score development in preschool children

	β estimate^a^	95% CI	*p*-value^b^	QIC^c^
Entire cohort (*n* = 491)				
Worries	0.14	0.01 – 0.27	0.039	2051.99
Tension	0.19	0.07 – 0.31	0.003	2041.97
Lack of Joy	0.15	0.04 – 0.27	0.009	2045.80
Demands	0.19	0.05 – 0.33	0.006	2048.45
Girls only (*n* = 241)				
Worries	0.24	0.06 – 0.41	0.009	1005.93
Tension	0.22	0.05 – 0.39	0.014	1013.26
Lack of Joy	0.23	0.08 – 0.38	0.002	1003.31
Demands	0.24	0.05 – 0.43	0.012	1012.31
Boys only (*n* = 250)				
Worries	-0.01	-0.18 – 0.18	0.963	1056.08
Tension	0.13	-0.03 – 0.29	0.108	1048.00
Lack of Joy	0.06	-0.11 – 0.23	0.503	1054.36
Demands	0.11	-0.08 – 0.31	0.247	1053.93

^a^Estimates derived from general estimation equations (GEE) for BMI z-scores (birth to age 5) as dependent variable, adjusted for gestational week at delivery, mode of delivery, pregnancy cotinine levels and breastfeeding duration

^b^Bonferroni adjusted significance level, *p* ≤ 0.0125

^c^Quasi- Akaike Information Criterion *QIC* for model selection

### Influence of maternal stress on early maternal feeding behavior

The strong association of maternal perceived stress on BMI z-scores during the first year of life suggested a possible involvement of breastfeeding duration or the time of solid food introduction. However, both parameters, evaluated in three-month-intervals during the first year of life, were not related to pre- and postnatal maternal perceived stress in our analyses (Additional file 1: Table S6).

### Multiple stressors contribute to maternal stress perception

**Fig.1:**
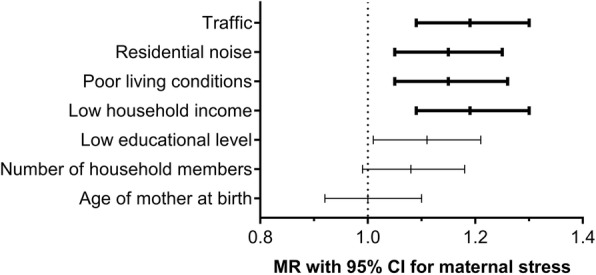
Effects of different stressors on maternal perceived stress. Mean ratios and 95 % confidence intervals for the effect of shown stressors on maternal perceived stress levels at year 1 were calculated from multiple regression models. Significant associations after Bonferroni correction are depicted in bold, *p* ≤ 0.007

Based on a 10-items questionnaire assessing the neighborhood quality (Additional file 1: Table S1), three factors, which in combination explained 58.1% of the variance (Additional file 1: Table S7), were extracted. The items that clustered on the same factor loadings suggest that the factors represent “poor living conditions”, burdens due to “traffic”, and exposure to “residential noise”, respectively (Additional file 1: Table S7). For each of these three factors corresponding variables were created including items with a factor loading >0.4. The variable “poor living conditions” considered the occurrence of vandalism, graffities, dirty streets and attempted break-ins in the living environment. The impairment due to “traffic” was summarized by items asking for disturbance due to traffic noise or related odors or exhausts. The variable “Noise” took into account noise from pedestrians and neighbors. All three of these variables showed a significant association to the maternal stress level at year one (Fig. 1).

Of the socio-demographic factors analyzed, only low household income contributed significantly to the overall stress perceived by the mothers (Fig. 1, Additional file 1: Table S8). Parental separation or divorce during the first three years (available from 191 participants) significantly increased the total maternal stress level at year 1 and three of the four different dimensions thereof (Additional file 1: Table S9).

### Associations of different stressors on stress dimensions

While “worries”, “tension”, and “demands” were similarly positively associated with the factors of poor neighborhood quality, “lack of joy” was not affected (Additional file 1: Figure S1A). However, “lack of joy” was positively associated with a “low household income”, as was an increase in “worries”. A low educational level and the number of household members, which were not associated to the overall maternal stress level, were significantly associated with the stress dimensions “lack of joy” and “demands”.

### Impact of stressors on BMI z-score development

To further elucidate how the stressors, which showed a significant association with the maternal perceived stress level at year 1 (see Additional file 1: Table S8), namely noise, traffic, poor living environment) and a low household income affect the BMI z-score development we assessed their relationship by mediation analyses. These stressors did not affect girl’s BMI z-score development directly. However, they had an indirect effect on BMI z-scores mediated by maternal perceived stress (Additional file 1: Figure S1B). Due to the small case number the effect of divorce/separation could not be further evaluated in the mediation analysis.

## Discussion

Obesity is a multifactorial disease with an often protracted onset in childhood and adolescence. Despite the appreciation that high caloric intake and sedentary behavior contribute to overweight development, less attention has been given to the effects of pre- and postnatal perceived maternal stress on weight development in early childhood. Given the few longitudinal studies evaluating this relationship in children [8, 39, 42, 43] our aim was to investigate whether and how maternal stress assessed at different time points in the highly vulnerable early pre- and postnatal period is related to overweight development in preschool children.

Previous studies have suggested that dramatic life events during the prenatal phase, such as the occurrence of a natural disaster or the death of a close relative, can contribute to overweight development in preschool children or in young teenagers [44–46]. In our study we did not observe a longitudinal association of prenatal stress on BMI z-scores but rather identified the perceived stressfulness for the mother during the first year of life as an important factor for an increase in BMI z-scores. This observation is in line with a recent meta-analysis by *Tate et al.,* suggesting that toddlers (1-3 years old) are more vulnerable to maternal stress than infants (<one year) [12]. In addition, as the type of stress evaluated in our study is most likely not comparable in its impact to a natural disaster, the missing association between prenatal perceived stress and BMI z-scores might also at least in part be related to the difference in the type of stress evaluated.

As only maternal stress during the first year after birth had an effect on BMI trajectories we hypothesized that duration of breast feeding, which can be negatively affected by maternal stress, might play a role [47]. However, we did not observe significant differences in breastfeeding duration or the time of solid food introduction in relation to the maternal stress level. This does not exclude the possibility that maternal feeding styles and attitudes may play a role [48, 49] as earlier studies indicate that the parent-created environment can foster obesity-promoting feeding styles and attitudes, which shape the child’s food preferences [50, 51]. In older children however, other social influences and food environments experienced in daycare or preschool may dilute the maternal stress effect. Although this is in line with our observation that only maternal perceived stress during the first year but not thereafter had a persistent effect on BMI z-scores, the design of our study did not allow further evaluation of parental feeding styles and attitudes.

Although high maternal stress levels were associated with higher BMI z-scores in the total LINA cohort, this effect was only present in girls, whereas boys were not affected. Similar observations were made by *Suglia et al.* [39], who described a higher risk of being obese for five-year-old girls, who had experienced high cumulative stress (including food insecurity, housing insecurity, maternal depressive symptoms, and maternal substance abuse) compared to girls without this experience, a similar effect was missing in boys [39].

In adults and adolescents gender disparity in stress perception and processing has previously been associated to differences in coping mechanisms [52–54]. Children seem to respond in a similar way as in particular girls have been described to respond by impulsive eating, emotional binge eating, and by requesting sweet and high fat foods [25, 39, 55]. In light of these previous findings, our results suggest that already at a very young age changes in eating behavior might play a role in the gender-disparity of BMI-development related to the experience of stress.

In accordance with what we saw for the overall maternal perceived stress, “demands”, “worries” and “lack of joy” had a strong positive association with BMI z-scores in girls only. There are several studies suggesting that maternal depression can promote overweight development in children [56, 57], with indications that this might also be a gender-specific effect [9], e.g. *Hernandez et al.* reported that maternal depression placed females but not males at a higher risk for obesity at age 18 [58].

On a last scale of our analyses we aimed to characterize potential stressors, which add to the perceived maternal stress level throughout the years. We were able to identify three potential stressors, which were all related to the quality of the living environment (burden due to traffic, residential noise, and poor living conditions) contributing to the overall maternal perceived stress.

We show in this study that different sources of noise including “Residential Noise” - noise from neighbors and pedestrians - and “Traffic” summarizing traffic noise and exhaust, can affect the maternal stress level. Noise exposure is a well-described example of a potentially obesogenic factor, which has already been studied for its impact on prenatal/postnatal growth [59–61] and in association to adiposity and metabolic outcomes in adults [27, 29, 62]. Noise during pregnancy and childhood increased the risk of overweight at age 7, although no association to BMI z-scores was found in this study [63]. Also, an unsecure living environment has been suggested to contribute to overweight development. While *Mathis et al.* observed that adults who perceive their neighborhood as unsecure were 12 times more likely to be overweight [64], in children neighborhood crime was associated with an increase in weight and a limited outdoor activity [65]. Next to the quality of the living environment the only other stressor with a significant impact on overall perceived maternal stress was a low household income, These stressors are likely related to a low socioeconomic status of the families, which is known to be strongly associated with obesity in the Western world [66, 67].

Interestingly, the stressors themselves did not have a direct effect on girl’s BMI z-scores but rather mediated their effect through their impact on the maternal overall perceived stress level. As the maternal perceived stress is most likely a cumulative account of these stressors and potentially also covers others stress-related factors, single stressors might not have a sufficient predictive power. This is in line with the observation that a combination of adverse effects within a family can increase the obesity risk, whereas unfavorable social factors in isolation often do not [68, 69].

## Conclusion

In summary, with our longitudinal qualitative stress assessment we were able to provide evidence for the idea that childhood BMI trajectories develop early and that maternal stress during the first year after birth is a persistent positive predictor of BMI z-scores in girls up to the age of five years.

To reduce the risk for childhood obesity – in particular in girls - behavioral interventions to reduce the mental stress in mothers should be considered in the future.

## Additional file

Additional file 1: **Table S1.** Questionnaire for the assessment of the living environment. **Table S2.** Impact of maternal perceived stress levels during pregnancy, year 1 and year 2 on longitudinal BMI z-score development in preschool children (birth-age5). **Table S3.** Gender disparity in susceptibility to maternal stress-related BMI development in preschool children age 1-5 years. **Table S4.** Comparison of gender-related study characteristics of the analyzed sub-cohort. **Table S5.** Characteristics of maternal perceived stress scores of the four different stress dimensions at year 1. **Table S6.** Influence of maternal stress during the first year after birth on breastfeeding duration and introduction of solid food. **Table S7.** Summary of exploratory factor analysis results of questionnaire items assessing the living environment. **Table S8.** Association of different stressors with the maternal stress levels at year 1. **Table S9.** Contribution of separation or divorce on perceived maternal stress at year 1. **Figure S1.** (A) Associations of different stressors and the four different stress dimensions at year 1. (B) Summary of mediation analysis. (DOCX 241 kb)

## Abbreviations

BMI: Body mass index
GEE: Generalized estimating equation
PSQ: Perceived Stress Questionnaire
WHO: World Health Organization
