# Supplementary material for: Early maternal perceived stress and children’s BMI: longitudinal impact and influencing factors
Source: BMC Public Health. 2018 Oct 30;18:1211. doi: 10.1186/s12889-018-6110-5 (PMC6208039; doi:10.1186/s12889-018-6110-5)
Supplement: Supplementary file 1 — Table S1. Questionnaire for the assessment of the living environment. Table S2. Impact of maternal perceived stress levels during pregnancy, year 1 and year 2 on longitudinal BMI z-score development in preschool children (birth-age5). Table S3. Gender disparity in susceptibility to maternal stress-related BMI development in preschool children age 1-5 years. Table S4. Comparison of gender-related study characteristics of the analyzed sub-cohort. Table S5. Characteristics of maternal perceived stress scores of the four different stress dimensions at year 1. Table S6. Influence of maternal stress during the first year after birth on breastfeeding duration and introduction of solid food. Table S7. Summary of exploratory factor analysis results of questionnaire items assessing the living environment. Table S8. Association of different stressors with the maternal stress levels at year 1. Table S9. Contribution of separation or divorce on perceived maternal stress at year 1. Figure S1. (A) Associations of different stressors and the four different stress dimensions at year 1. (B) Summary of mediation analysis. (DOCX 241 kb) [file 12889_2018_6110_MOESM1_ESM.docx]

**Supplementary Material**

**Early maternal perceived stress and children’s BMI: longitudinal impact and influencing factors**

Beate Leppert*& Kristin Junge*, Stefan Röder, Michael Borte, Gabriele I. Stangl, Rosalind J. Wright, Anja Hilbert, Irina Lehmann* & Saskia Trump*

*authors contributed equally

**Content**

Table S1: Questionnaire for the assessment of the living environment.

Table S2: Impact of maternal perceived stress levels during pregnancy, year 1 and year 2 on longitudinal BMI z-score development in preschool children (birth-age5).

Table S3: Gender disparity in susceptibility to maternal stress-related BMI development in preschool children age 1-5 years.

Table S4: Comparison of gender-related study characteristics of the analyzed sub-cohort.

Table S5. Characteristics of maternal perceived stress scores of the four different stress dimensions at year 1.

Table S6. Influence of maternal stress during the first year after birth on breastfeeding duration and introduction of solid food.

Table S7: Summary of exploratory factor analysis results of questionnaire items assessing the living environment.

Table S8: Association of different stressors with the maternal stress levels at year 1.

Table S9: Contribution of separation or divorce on perceived maternal stress at year 1.

Figure S1: (A) Associations of different stressors and the four different stress dimensions at year 1. (B) Summary of mediation analysis.

**Table S1: Questionnaire for the assessment of the living environment.**

| **How often did you experience the following impairments in your neighborhood?**  *(just one cross in each line)* | | | | |
| --- | --- | --- | --- | --- |
|  | **hardly ever**  (1) | **sometimes**  (2) | **often**  (3) | **usually**  (4) |
| Traffic noise |  |  |  |  |
| Commercial noise |  |  |  |  |
| Odors and exhausts |  |  |  |  |
| Noise from neighbors |  |  |  |  |
| Noise from pedestrians |  |  |  |  |
| Noise from restaurants/clubs |  |  |  |  |
| Graffiti |  |  |  |  |
| Vandalism |  |  |  |  |
| Dirty streets (e.g. waste, dog excrements) |  |  |  |  |
| Attempted break-ins/thefts |  |  |  |  |

Answers were scored as indicated from 1 (hardly ever) to 4 (usually) to calculate a score for exposure to poor living conditions, traffic and residential noise.

**Table S2: Impact of maternal perceived stress levels during pregnancy, year 1 and year 2 on longitudinal BMI z-score development in preschool children (birth-age5).**

|  |  | **β estimate** _a_ | **95% CI** | ***p*-value** |
| --- | --- | --- | --- | --- |
| Pregnancy | (n=498) | 0.06 | -0.07 – 0.20 | 0.372 |
| Year 1 | (n=491) | **0.23** | **0.08 – 0.37** | **0.002** |
| Year 2 | (n=473) | 0.09 | -0.71 – 0.24 | 0.283 |
| Pregnancy to year 2 | (n=473) | 0.06 | -0.01 – 1.21 | 0.078 |

a - Estimates derived from general estimation equations (GEE) for BMI z-scores (birth to age 5) as dependent variable, adjusted for gestational week at delivery, mode of delivery, pregnancy cotinine levels and breastfeeding duration (not for pregnancy stress levels).

**Table S3: Gender disparity in susceptibility to maternal stress-related BMI development in preschool children age 1-5 years.**

|  | **β estimate** _a_ | **95% CI** | ***p*-value** |
| --- | --- | --- | --- |
| **Entire cohort** (n=491) |  |  |  |
| Raw | **0.22** | **0.08 – 0.37** | **0.003** |
| adj._b_ | **0.23** | **0.08 – 0.37** | **0.002** |
| **Girls only** (n=241) |  |  |  |
| Raw | **0.30** | **0.11 – 0.49** | **0.002** |
| adj. _b_ | **0.30** | **0.11 – 0.49** | **0.002** |
| **Boys only** (n=250) |  |  |  |
| Raw | 0.14 | -0.07 – 0.35 | 0.194 |
| adj. _b_ | 0.10 | -0.11 – 0.31 | 0.333 |

a - Estimates derived from general estimation equations (GEE) for BMI z-scores (birth to age 5) as dependent and maternal stress during the first year of life as independent variable.

b - Adjusted for gestational week at delivery, mode of delivery, pregnancy cotinine levels and breastfeeding duration.

**Table S4: Comparison of gender-related study characteristics of the analyzed sub-cohort.**

|  | **girls only**  n (%), n=245 _a_ | **boys only**  n (%), n=253 _a_ | **χ^2^-test** |
| --- | --- | --- | --- |
| **Week of gestation at birth** |  |  | 0.747 |
| <37 weeks | 7 (2.9) | 9 (3.6) |  |
| 37-40 weeks | 158 (64.5) | 150 (59.3) |  |
| >40 weeks | 80 (32.6) | 94 (37.1) |  |
| **Mode of delivery** |  |  | 0.962 |
| spontaneous | 191 (80.0) | 196 (77.5) |  |
| C-section | 51 (20.8) | 53 (20.9) |  |
| others | 3 (1.2) | 4 (1.6) |  |
| **Birth weight** |  |  | 0.35 |
| <3000g | 51 (20.8) | 41 (16.2) |  |
| ≥3000g – 3500g | 104 (42.4) | 91 (36.0) |  |
| ≥3500g – 4000g | 69 (28.2) | 82 (32.4) |  |
| ≥4000g | 21 (8.6) | 39 (15.4) |  |
| **Household members** |  |  | 0.871 |
| 2 | 12 (4.9) | 14 (5.6) |  |
| 3 | 152 (62.3) | 148 (58.7) |  |
| ≥4 | 80 (32.8) | 90 (35.7) |  |
| **Breastfeeding** |  |  | 0.958 |
| 1. -3. Month | 43 (18.5) | 44 (17.8) |  |
| 1.-6. Month | 78 (33.6) | 88 (35.6) |  |
| 1.-12. Month | 111 (47.8) | 115 (46.6) |  |
| **Education** _b_ |  |  | 0.822 |
| Low | 4 (1.6) | 2 (0.8) |  |
| Medium | 52 (21.2) | 49 (19.4) |  |
| High | 189 (77.1) | 202 (79.8) |  |
| **Household income** |  |  | 0.844 |
| <2000€ | 83 (36.1) | 89 (37.2) |  |
| 2000€ - 4000€ | 132 (57.4) | 130 (54.4) |  |
| >4000€ | 15 (6.5) | 20 (8.4) |  |
| **Separation/divorce** _c_ |  |  | 0.832 |
| Yes | 11 (12.2) | 12 (13.2) |  |
| No | 79 (87.8) | 79 (86.8) |  |
| **Pregnancy cotinine level** _d_ |  |  | 0.567 _e_ |
| median [µg/g creatinine] | 1.88 | 1.80 |  |
| <25%, >75% | 0.72, 5.13 | 0.80, 4.82 |  |

a – n may be different from total n due to missing data.

b – Low = 8 yrs of schooling (‘Hauptschulabschluss`); medium = 10 yrs of schooling (`Mittlere Reife`); high = 12 yrs of schooling or more (`(Fach-)hochschulreife’)..

c – Parental separation/divorce in the last 3 years from children’s age 3 years

d – ETS = environmental tobacco smoke (urinary cotinine level at pregnancy).

e – *p*-value derived from Student’s T-test between group means.

**Table S5: Characteristics of maternal perceived stress scores of the four different stress dimensions at year 1.**

|  | **median** | **min** | **max** | **<25%** | **>75%** |
| --- | --- | --- | --- | --- | --- |
| Worries | 1.60 | 1.00 | 4.00 | 1.20 | 2.00 |
| Tension | 2.20 | 1.00 | 4.00 | 1.80 | 2.60 |
| Lack of joy | 2.00 | 0.80 | 3.80 | 1.60 | 2.40 |
| Demands | 2.20 | 1.00 | 4.00 | 1.80 | 2.60 |

Stress dimensions were assessed with the reduced perceived stress questionnaire (PSQ) scored 1 (almost never) to 4 (usually) with 5 questions each, n=498.

**Table S6: Influence of maternal stress during the first year after birth on breastfeeding duration and introduction of solid food.**

|  | **β estimate** _a_ | **95% CI** | ***p*-value** |
| --- | --- | --- | --- |
| Breastfeeding duration _b_ | -0.01 | -0.10 – 0.08 | 0.798 |
| Introduction to solid food _b_ | -0.03 | -0.15 – 0.09 | 0.622 |

a - Estimates derived from general estimation equations (GEE) for BMI z-scores (age 1 to age 5) as dependent variable.

b - Breastfeeding and introduction to solid food was assessed in 3-month-intervals during the first year of life.

**Table S7: Summary of results of questionnaire items assessing the living environment by the question “How often did you experience the following impairments in your neighborhood?" (n=495).**

|  | **Rotated Factor Loadings** | | |
| --- | --- | --- | --- |
| **ITEMS** | Poor living conditions | Traffic | Residential Noise |
| Vandalism | **0.828** | -0.026 | 0.023 |
| Graffiti | **0.826** | -0.091 | 0.008 |
| Attempted break-ins/thefts | **0.479** | 0.091 | -0.078 |
| Dirty streets (e.g. waste, dog excrements) | **0.453** | 0.147 | 0.175 |
|  |  |  |  |
| Odors/exhausts | 0.001 | **0.797** | -0.028 |
| Traffic noise | 0.009 | **0.793** | 0.002 |
|  |  |  |  |
| Commercial noise | 0.006 | 0.362 | 0.080 |
| Noise from pedestrians | -0.091 | 0.002 | **0.885** |
| Noise from neighbours | 0.116 | 0.016 | **0.417** |
| Noise from restaurants/clubs | 0.042 | 0.018 | 0.176 |
| Eigenvalues | 3.35 | 1.43 | 1.03 |
| % of variance | 33.5 | 14.3 | 10.3 |
| α | 0.74 | 0.76 | 0.57 |

a - Factor loadings over 0.4 appear in bold.

b – Answered on a four-point scale from hardly ever (0) to usually (3).

**Table S8: Association of different stressors with the maternal stress levels at year 1. (n=491).**

|  | **Mean Ratio**_a_ | **95% CI**_a_ | ***p*-value_b_** |
| --- | --- | --- | --- |
| Traffic | **1.17** | **1.07 – 1.28** | **0,0003** |
| Residential Noise | **1.15** | **1.05 - 1.26** | **0.0020** |
| Poor Living Conditions | **1.14** | **1.054– 1.25** | **0.0034** |
| Low Household Income^c^ | **1.18** | **1.08 – 1.30** | **0,0003** |
| Low Educational Level | 1.10 | 1.01 – 1.21 | 0.032 |
| Number of Household Members | 1.07 | 0.97 – 1.17 | 0.156 |
| Age of the Mother at Birth | 0.98 | 0.90 – 1.07 | 0.671 |

a – Standardized mean ratios and confidence intervals (95% CI) derived from linear regression.

b - Bonferroni adjusted significance level, *p* ≤ 0.007.

c – n=462

**Table S9: Contribution of separation or divorce on perceived maternal stress at year 1 (n=191).**

| dependent variable | **β estimate**_a_ | **95% CI** | ***p*-value_b_** |
| --- | --- | --- | --- |
| Total maternal stress | **0.33** | **0.12 – 0.54** | **0.002** |
| Worries | **0.40** | **0.17 - 0.63** | **0.001** |
| Tension | 0.27 | 0.02 - 0.53 | 0.039 |
| Demands | **0.31** | **0.08 – 0.54** | **0.008** |
| Lack of Joy | **0.33** | **0.09 - 0.58** | **0.008** |

a – Unstandardized estimates derived from linear regression.

b – Bonferroni corrected significance level for two-sided p ≤ 0.01

**
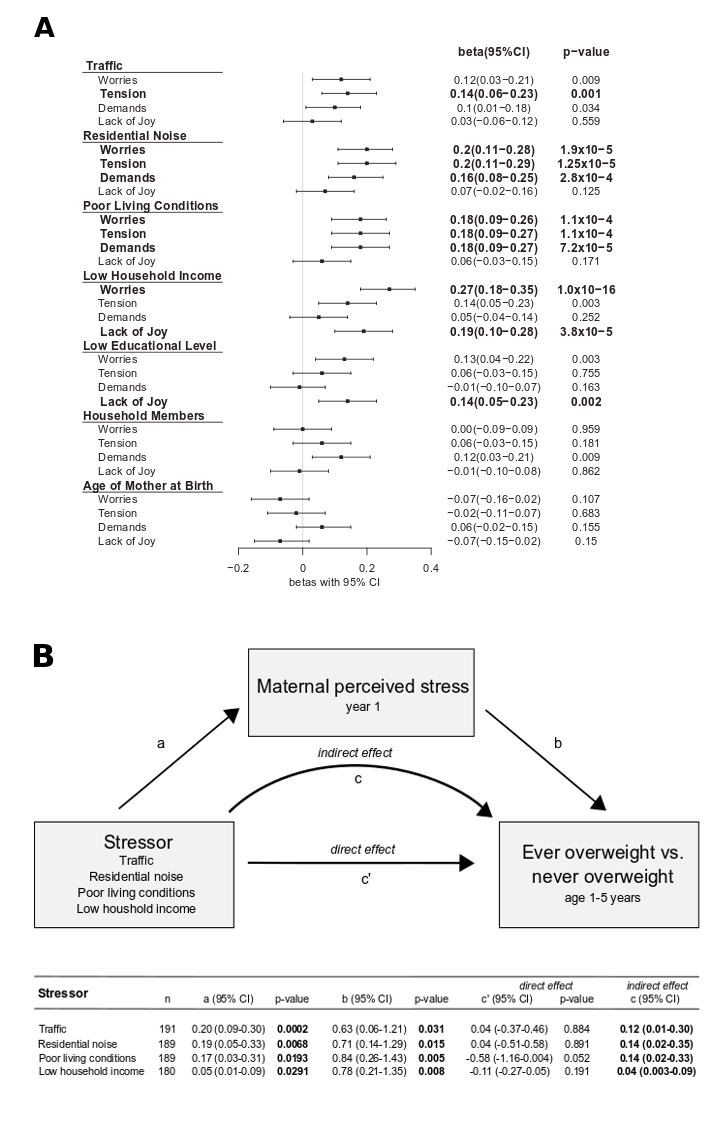
**

**Figure S1: Associations of different stressors and the four different stress dimensions at year 1 (n=489).** Shown are forest plots based on β values with 95% confidence intervals. Significant associations are depicted in bold. (B) **Summary of mediation analysis**. Stressors themselves had no direct effect on girl’s BMI-z-scores but rather mediated their effect by their contribution to the maternal perceived stress level. Logistic regression for path b was adjusted for gestational week at birth, mode of birth and smoking during pregnancy. Confidence intervals for the indirect effects were derived from 5000 bootstrap samples. Confidence intervals, which do not cross 0 indicate significance of the indirect effect.
